# Global loss of Neuron‐specific gene 1 causes alterations in motor coordination, increased anxiety, and diurnal hyperactivity in male mice

**DOI:** 10.1111/gbb.12816

**Published:** 2022-05-16

**Authors:** Roman Austin, Praveen Chander, Amber J. Zimmerman, Malene Overby, Laura Digilio, Chan Choo Yap, David N. Linsenbardt, Heidi Kaastrup Müller, Jason P. Weick

**Affiliations:** ^1^ Department of Neurosciences University of New Mexico School of Medicine Albuquerque New Mexico USA; ^2^ Department of Clinical Medicine, Translational Neuropsychiatry Unit Aarhus University Aarhus C Denmark; ^3^ Department of Cell Biology University of Virginia Charlottesville Virginia USA

**Keywords:** AMPA receptors, Calcyon, neuronal endosomal‐enriched protein 21 kDa (NEEP21), NSG2, synaptic plasticity

## Abstract

The Neuron‐specific gene family (NSG1‐3) consists of small endolysosomal proteins that are critical for trafficking multiple receptors and signaling molecules in neurons. NSG1 has been shown to play a critical role in AMPAR recycling from endosomes to plasma membrane during synaptic plasticity. However, to date nothing is known about whether NSG1 is required for normal behavior at an organismal level. Here we performed a battery of behavioral tests to determine whether loss of NSG1 would affect motor, cognitive, and/or affective behaviors, as well as circadian‐related activity. Consistent with unique cerebellar expression of NSG1 among family members, we found that NSG1 was obligatory for motor coordination but not for gross motor function or learning. NSG1 knockout (KO) also altered performance across other behavioral modalities including anxiety‐related and diurnal activity paradigms. Surprisingly, NSG1 KO did not cause significant impairments across all tasks within a given modality, but had specific effects within each modality. For instance, we found increases in anxiety‐related behaviors in tasks with multiple stressors (e.g., elevation and exposure), but not those with a single main stressor (e.g., exposure). Interestingly, NSG1 KO animals displayed a significant increase in locomotor activity during subjective daytime, suggesting a possible impact on diurnal activity rhythms or vigilance. Surprisingly, loss of NSG1 had no effect on hippocampal‐dependent learning despite previous studies showing deficits in CA1 long‐term potentiation. Together, these findings do not support a role of NSG1 in hippocampal‐dependent learning, but support a role in mediating proper neuronal function across amygdalar and cerebellar circuits.

## Graphical abstract

Neuron‐specific gene family member 1 (NSG1) is a small endolysosomal protein that is critical for trafficking multiple receptors and signaling molecules in neurons. Here we demonstrate that NSG1 KO animals show gait abnormalities, consistent with unique NSG expression in Cerebellum. NSG1 KO also caused increased anxiety in elevated mazes, and hyperactivity during subjective daytime, suggesting more global effects on cortical and subcortical networks.

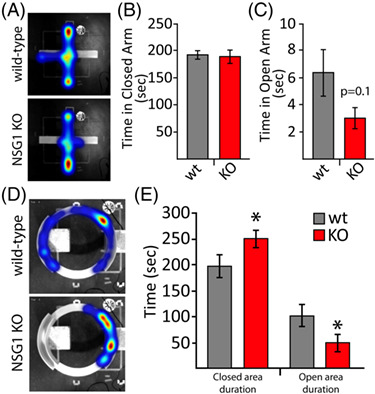

## 1 INTRODUCTION

NSG family members are small, brain‐enriched proteins that arose specifically during the evolution of the vertebrate clade^1^ suggesting a potential role in more advanced cognitive, emotional and/or motor behaviors. Neuron‐specific gene 1 (NSG1; NEEP21) is a 21 kDa, single transmembrane‐containing protein critical for trafficking multiple cargo proteins through the secretory and endolysosomal system, exclusively in neurons. NSG1‐dependent cargos include L1 cell adhesion molecule (L1CAM),^2^ Neurotensin receptors 1–2,^3^ as well as amyloid‐precursor protein (APP).^4^ However, the role of NSG1 in activity‐dependent AMPAR trafficking during synaptic plasticity is the most well‐characterized. NSG1 forms a multi‐molecular complex involving interactions between GRIP1, GLUA2, and Syntaxin 13.^5^ Antisense‐mediated down‐regulation of NSG1 impedes recycling of GluA1 and GluA2 in hippocampal neurons following treatment with NMDA.^5^ NSG1 knockdown, or expression of a dominant negative NSG1 fragment (aa129‐164), reduces both miniature AMPAR‐mediated excitatory postsynaptic currents (mEPSCs) as well as evoked currents. Further, disruption of NSG1 function causes reductions in the degree of long‐term potentiation (LTP) in response to high frequency stimulation in organotypic hippocampal slices.^6^ Together, these findings strongly support NSG1 is a critical regulator of neuronal function and plasticity.

While NSG1 is expressed widely throughout the brain,^7^ the role of NSG1 in specific neural circuits remains unknown. NSG1 is one of three members of the Neuron‐specific gene family (NSG), the others being NSG2 (P19) and NSG3 (Calcyon, Caly).^8^ While less is known about NSG2,^9^, ^10^ NSG3/Caly has been well characterized, and behavioral studies may provide clues as to the brain circuits regulated by other NSG family members. In contrast to NSG1, NSG3/Calcyon is critical for clathrin‐mediated *endocytosis* of AMPARs, where overexpression *reduces* AMPAR surface expression and knockout (KO) impairs long‐term depression (LTD; [^11^, ^12^, ^13^, ^14^]). Overexpression (OE) of NSG3 causes hyperactivity when exploring an open field apparatus in addition to stereotypies, rearing and sniffing behaviors.^15^ NSG3 OE animals also display reduced anxiety, spending more time in the light areas of a light–dark box and open areas of the elevated plus maze.^15^ Further, NSG3 OE causes reductions in response inhibition during the extinction phase of context‐dependent fear conditioning, despite normal acquisition of learning on this task.^13^ Furthermore, NSG3 OE also caused significant perseverative errors on a version of the Morris Water Maze task which required animals to flexibly learn a new platform location following acquisition of an initial location. While NSG3 OE animals could do this, probe trials revealed that OE animals spent far more time searching for the initial platform location, suggesting long‐term inflexible behavior.^13^

Thus, behavioral analysis of NSG1 KO animals could provide significant insight into how NSG1 regulates neuronal circuits, and possibly complements that of other NSG family members. Based on its relatively overlapping expression patterns with other NSG family members,^7^, ^16^ KO of NSG1 might be predicted to display impaired hippocampal‐dependent learning and memory similar to NSG3 KO. However, NSG1 displays a broad distribution throughout excitatory neurons of the cerebral cortex in addition to hippocampal expression. Further, NSG1 is unique among NSG family members in its enrichment in Purkinje neurons of the cerebellum.^7^ Furthermore, given the multifaceted role of NSG1 in trafficking multiple cargos critical for the development and maintenance of excitatory circuits, alterations in NSG1 levels may have wide‐ranging effects on cognitive, affective, and even motor function. However, no studies to date have tested the impact of NSG1 KO at the behavioral level. Thus, we undertook a series of behavioral tests on a newly generated NSG1 KO animal (see Section 2) to survey the impact of loss of NSG1 across motor, anxiety, and cognitive domains. Surprisingly, we found that NSG1 KO animals displayed behavioral deficits across multiple motor and anxiety‐related domains, but were not impaired in hippocampal‐based learning and memory.

## 2 MATERIALS AND METHODS

All experimental procedures described here adhered to the US Public Health Service policy on humane care and use of laboratory animals and were approved by the Institutional Animal Care and Use Committee of both the University of Virginia and the University of New Mexico Health Sciences Center. For all the experiments described in the following sections, the experimenters were blinded to the treatment group assignment.

### 2.1 Generation of NSG1 mutant mice

NSG1 KO mice were generated as follows: cryopreserved sperm from the strain Nsg1^tm1.1(KOMP)Vlcg^ was obtained from the KOMP repository and IVF was performed by the Genetically Engineered Murine Model (GEMM) Core at UVA. The sperm and females used for IVF were from the C57BL/6N strain. Heterozygotes resulting from IVF were bred to produce the NSG1 KO. Offspring were weaned at approximately 21 days of age, ear tagged, and housed with same sex littermates at 22°C on a 12‐h reverse light/dark cycle. The mice had free access to standard chow and water in their cages. All experimental mice were sex‐matched progeny of pairings between heterozygous (NSG1^+/−^) C57BL6/N mice. Behavior tests were performed on two different cohorts of 12 wild‐type and KO male mice (total *n* = 24). The mice across cohorts were born within 2 weeks of each other and were tested at the same ages (e.g., 2‐weeks apart). Behavior was tested on mice between the ages of 2 and 6 months. All of the experiments were performed during the active phase between the hours of 9–17 (3–11 h into the dark cycle), in a room illuminated by red lights, except for homecage behavioral monitoring, which occurred during both light and dark phases. Behavioral assays were performed in the following order: open field, rotarod, Catwalk XT, elevated plus maze, elevated zero maze (EZM), trace fear conditioning, homecage monitoring. All tests were separated by a 24–48 intertest interval.

### 2.2 NSG1 genotype validation: qPCR‐based genotyping

Details of the gene targeting strategy for the NSG1 KO mice can be found in reference 7. Tail snips (0.5 cm) were digested in 200 μl of DirectPCR Lysis Reagent (Viagen Biotech, Los Angeles, CA) and 2 μl of Proteinase K (Zymo Research, Irvine, CA) at 55°C for 24 h followed by incubation at 85°C for 45 min to inactivate Proteinase K. Polymerase chain reaction (PCR) amplification of NSG1 was carried out using Q5 DNA polymerase according to the manufacturer's recommendations (New England Biolabs, Ipswich, MA). The following primers were used to determine specific allelic expression: ACTTGCTTTAAAAAACCTCCCACA (LacZ forward), TCCAGAGTGAGTGAGACATGGAAGC (NSG1 wild‐type forward), AGAGTAGGGTGCAATGACCAAGAGG (NSG1 wild‐type reverse), and CCAAAGAGAACCTCCCAGAATTGCC (NSG1 KO reverse). 1 μl of digested genomic DNA was used in a 25 μl PCR reaction containing all four primers (5 μM each). The following cycling parameters were used: 1 cycle of 94°C for 5 min, 10 cycles of 94°C (15 s), 65°C (30 s), 72°C (40 s) with a decrease of 1°C per cycle for 10 cycles, 30 cycles of 94°C (15 s), 55°C (30 s), 72°C (40 s), and 1 cycle of 72°C for 5 min (C1000 Touch Thermal Cycler, Bio‐Rad, Hercules, CA). 2–5 μl of the PCR reactions were subjected to gel electrophoresis using a 2% agarose gel in tris‐acetate‐ethylenediamine tetraacetic acid buffer. Amplicons were visualized and compared with a 100 bp DNA ladder to identify mice with wild‐type (309 bp), KO (844 bp), and heterozygote (309 and 844 bp) genotypes.

### 2.3 Western blot

Brains from wild‐type and NSG1 KO mice were homogenized in 5 mM CHAPS, 50 mM Tris–HCl, 150 mM NaCl, 1× proteinase inhibitor with a Kontes microtube pellet pestle while kept on ice. Detergent‐insoluble material was removed by centrifugation at 13,000× g for 10 min. Aliquots of supernatants were mixed with SDS sample buffer (125 mM Tris–HCl, pH 6.8, 20% glycerol, 4% SDS, 0.02% bromophenol blue, and 125 mM dithiothreitol) and incubated at 50°C for 20 min. The samples were separated on a 10% NuPage Bis‐Tris gel (Invitrogen) using a MES buffer system (Invitrogen), transferred to nitrocellulose membranes using the Trans‐Blot Turbo Transfer System (Bio‐Rad), blocked in Odyssey Blocking Buffer (LI‐COR), and probed simultaneously with the primary antibodies: mouse anti‐NSG1 (Santa Cruz; sc‐390654, 1:1000) and rabbit anti‐NSG2 (Abcam; ab189513, 1:1000) followed by incubation with the IRDye conjugated secondary antibodies: goat antimouse800CW (LI‐COR; 926‐32210, 1:10,000) and goat antirabbit680RD (LI‐COR; 926‐68071, 1:10,000). The Odyssey CLx infrared imaging equipment was used to detect infrared signals.

### 2.4 Immunohistochemistry

Wild‐type and NSG1 KO mice were anesthetized and perfused with phosphate buffered saline (PBS) followed by 4% paraformaldehyde (PFA). Brains were removed and immersed in 4% PFA, 20% sucrose, and 30% sucrose, each for 12–24 h. Sagittal sections were sliced on a sliding knife microtome (American Optical). Free‐floating sections were permeablized and blocked simultaneously in 0.5% Triton X‐100 (Sigma‐Aldrich, St. Louis, MO) and 10% donkey serum (Millipore‐Sigma) for 1 h in PBS. Sections were stained with goat anti‐NSG1 (Thermofisher; PA5‐37939, 1:1000) rabbit anti‐NSG2 (Abcam; ab189513, 1:500) in 0.25% Triton and 5% donkey serum in PBS overnight at 4°C. Sections were washed three times in PBS followed by secondary antibody labeling (donkey antigoat and donkey antirabbit [1:1000]; Thermofisher) in the same buffer as primary antibodies. Sections were then mounted to microscopy slides (Superfrost plus, Fisher Scientific), immersed in Fluoromount‐G, and imaged on a slide‐scanning microscope (Zeiss Axion Scan.Z1) with Colibri 7 LED light source. Standard fluorescence calibration was performed on control brain sections to ensure proper dynamic range of signals and imaging conditions were maintained on NSG1 KO brains.

### 2.5 Anxiety‐related tasks

The open‐field test was used to assess exploratory behavior, anxiety, and gross locomotion according to previous methods.^17^ Briefly, the arena was an opaque square box measuring 60 x 60 x 42 cm^3^. Mice were placed in the middle of the arena and tracked for a period of 5 min. An overhead camera (Med Associates Basler acA1300‐60) Ethovision tracking software XT (Noldus Information Technology, Wageningen, Netherlands) measured velocity, distance traveled, and time spent in different locations within the arena. The chamber was cleaned using 70% ethanol between trials to eliminate olfactory distractions.

The elevated plus maze (EPM) tests the natural tendency of mice to avoid open and elevated areas.^18^, ^19^ Mice that are anxious spend relatively more time in the closed arms where they feel safe, whereas mice with lower levels of anxiety spend relatively more time in the open arms of the apparatus. The apparatus was elevated 65 cm above the ground and consisted of a central platform that intersects two open (60 x 7.6 cm^3^) and closed arms (60 x 7.6 x 24 cm^3^) positioned at 90° to each other. Each mouse was placed on the central platform and tracked for a period of 5 min using the Ethovision tracking software. For scoring purposes, animals were considered to be in a particular arm when all four paws were touching the floor of that arm. The chamber was cleaned using 70% (v/v) ethanol solution (Sigma‐Aldrich) between trials to eliminate olfactory distractions. The software measured latency and cumulative time spent on the open and closed arms, as well as the frequency and number of entries, which is used as an index of general activity.

The EZM^20^ was used to assess anxiety and exploratory behavior and is considered to be more anxiety specific than the open field test^21^ and may pick up on subtle motor differences compared with the EPM.^22^ The EZM was performed largely as described previously.^23^ The apparatus consisted of an elevated circular platform (64 cm high; 50.8 cm min diameter and 60.96 cm max diameter) with two opposing enclosed sections enclosed by a wall (20 cm high) and two opposing open sections, each with a platform 5 cm wide. Animal home cages were moved into the testing room illuminated at 90 lux approximately 2 h before testing. Each mouse was randomly placed in one quadrant within the maze and allowed to explore for 5 min. An overhead camera (Med Associates Basler acA1300‐60) and Ethovision X‐T video‐tracking software (Noldus) were used to monitor the position of the central point of the mouse body. The software measured latency and cumulative time spent in the open and closed areas of the maze. Between trials, the apparatus was cleaned with 70% ethanol and thoroughly dried.

### 2.6 Motor‐related tasks

The rotarod task was used to assess motor performance and fatigue resistance in rodents using the Panlab Rota Rod model LE8205. The rod was initiated to rotate at a constant initial speed of 4 RPM. Once the mouse was positioned, the rod accelerated to 40 RPM in 300 s. The time spent on the rod and the RPM reached at the time of falling was captured for each of five trials. A 30‐s intertrial interval (ITI) was used throughout the experiment. The chamber was cleaned using 70% ethanol between animals to eliminate olfactory distractions.

The CatwalkXT system (Noldus) was used to analyze gait and fine motor coordination according to methods previously described.^24^ Mice were placed at one end of a 1.3 m glass runway and allowed to walk across to the other end to reach a dark goal box. Footprint detection occurred as animals passed through a black tunnel illuminated from one side by a reflected green fluorescent light. Footprints were captured by a high‐speed camera across an area that was manually adjusted to capture at least three full stride‐lengths. Three trials were captured for each animal during one testing session. Trials were classified as being compliant by the software if the mouse crossed the recording area under 5 seconds and did not show a maximum speed variation greater than 60%. The glass plate was cleaned using 70% ethanol between mice to eliminate olfactory distractions. Compliant trials were analyzed via automatic detection but were also reviewed manually; if the mouse stopped or turned in mid‐run the trials were excluded. Analysis was performed using Catwalk XT 8.1 Software. Gait and movement were analyzed for right fore (RF), left fore (LF), right hind (RH) and left hind (LH) paws on the following variables: paw print area (size of paw print area during a full stance), stride length (distance between two consecutive paw placements of the same paw), swing (time interval between two consecutive paw placements of the same paw), and swing speed (velocity of an individual paw between two consecutive placements). The percentage of step patterns categorized as cruciate, alternate or rotary was analyzed as previously described.^25^

### 2.7 Learning and memory

Pavlovian trace fear conditioning (TFC) was used to measure associative learning using methods adapted from previous publications.^26^, ^27^ The apparatus was a 25 x 28 x 22 cm^3^ chamber with white walls and a metal rod floor (Med Associates NIR‐022SD). On the training day of the trace fear conditioning protocol, animals were placed into a Coulbourn Instruments (Allentown, PA) Habitest® System for 180 s to habituate to the apparatus. After a 120‐s no stimulus period, a 90 dB, 5000 Hz white noise auditory tone was presented for 30 s (conditioned stimulus [CS]) and followed by a 20‐s interval that terminated with a 2 second 0.7 mA footshock (unconditioned stimulus [US]). After the first tone/shock pairing there was a 90‐s no stimulus interval followed by another tone/shock pairing. The tone/shock pairings were repeated 5 times in total with a randomized interval averaging 120 s. Each session was ended 60 s after the final pairing. To eliminate olfactory distractions, the chamber was cleaned using 70% ethanol between trials.

Memory for the CS was tested absent the shock in a novel environmental context 24 h after conditioning. The novel context was a 25 x 28 x 22 cm^3^ chamber with black and white checkered walls and a different metal rod floor. The chamber was cleaned using 70% ethanol between trials to eliminate olfactory distractions. The protocol used was the same as the day prior without the US present.

### 2.8 Circadian regulation

Spontaneous homecage activity was collected in control and NSG1 KO mice to assess circadian cycles and locomotor behavior in a nonaversive environment. All mice were individually housed in a standard home cage with corncob bedding with ad libitum food and water and left undisturbed for a 72‐h period under their normal reverse light–dark conditions (lights on at 2000 h and off at 0800 h). Horizontal activity was automatically measured by photocell beam break for 72 h using the PAS‐Homecage system (San Diego Instruments, San Diego, CA). The first 9 h were included as an acclimation period, and the middle 48 h were used for data analysis (starting at 0800 h, light offset). Repeated measures ANOVA was performed across the entire circadian period and two‐way ANOVA was performed on cumulative light and dark activity across the 48‐h period to assess light (sleep) and dark (wake) phases separately.

### 2.9 Statistical analysis

For Homecage, TFC, and rotorod analyses we used repeated measures ANOVA with post‐hoc tests unless otherwise stated. For Catwalk XT, open field, EPM, and EZM we used multivariate ANOVA's with post‐hoc tests unless otherwise stated. For comparisons of distance traveled on open field, EPM, and EZM, unpaired *t*‐tests were used with statistical significance set a priori at *p* = 0.05.

## 3 RESULTS

### 3.1 Validation of NSG1 KO mice

**FIGURE 1:**
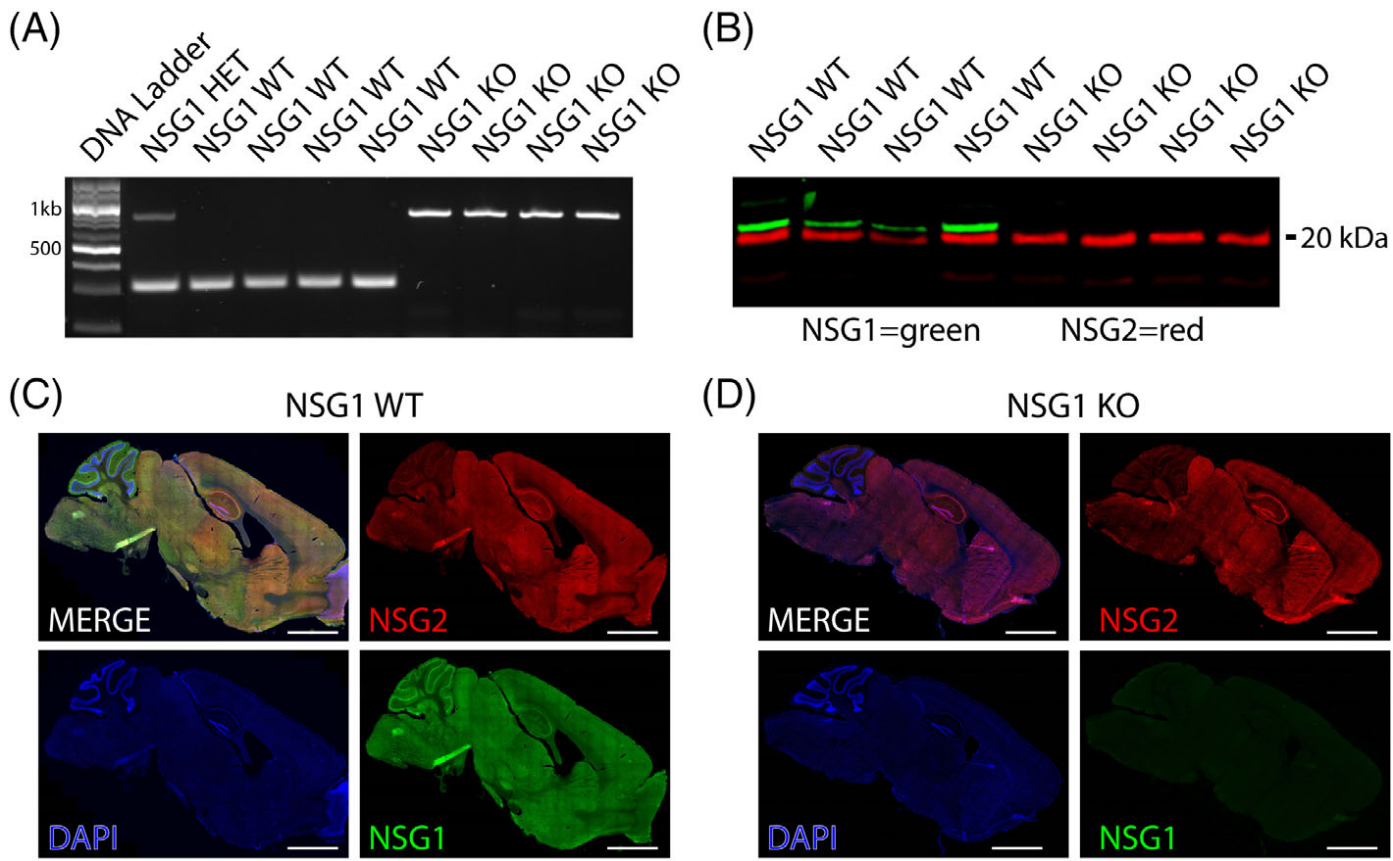
Validation of global NSG1 knockout (KO) in null animals. (A) Agarose gel showing PCR‐based genotype analysis of a NSG1 heterozygote (lane 2), four NSG1 wild‐type (lanes 3–6), and four NSG1 KO animals (lanes 7–10). DNA ladder (lane 1) is show for molecular weight reference. (B) Western blot illustrating labeling of NSG1 (green) and NSG2 (red) from four wild‐type (left‐most lanes) and four KO animals (right‐most lanes) used in behavioral testing. (C‐D) Immunohistochemical staining of NSG1 (green), NSG2 (red), and DAPI (blue) on parasagittal brain sections from a wild‐type (C) and KO animal (D). Note the robust cerebellar staining of NSG1 antibody in wild‐type brain section while NSG2 signal is relatively low, as well as lack of NSG1 signal in a section from an NSG1 KO animal (bottom right panel in D)

We first validated the loss of NSG1 protein in null animals. Figure 1A displays an agarose gel in which PCR amplicons of genomic DNA were visualized from an NSG1 heterozygote (lane 2), four NSG1 wild‐type (lanes 3–6), and four NSG1 KO animals (lanes 7–10). Loss of protein expression was first validated by western blot of brain extracts taken from four wild‐type and four NSG1 KO animals that were probed for NSG1 (Figure 1B, green, upper bands) and anti‐NSG2 (Figure 1B, red, lower bands). Wild‐type samples displayed robust staining for both proteins near 20 kDa (Figure 1B, left four lanes), while NSG1 KO samples showed positive staining for NSG2 only (Figure 1B, right four lanes). To demonstrate brain‐wide loss of NSG1 in KO animals we probed parasagittal sections of wild‐type (Figure 1C) and KO animals (Figure 1D). Wild‐type animals displayed similar expression patterns for both NSG1 (green) and NSG2 (red) across multiple brain regions. One major exception is the robust expression of NSG1 in cerebellar Purkinje neurons, with a near absence of NSG2 expression, consistent with previous findings.^7^ In contrast, sections from NSG1 KO animals showed complete absence of specific staining in all regions (Figure 1D, green lower right panel). Thus, the gene targeting strategy employed in Barford et al.^7^ specifically eliminated NSG1 protein expression throughout the brain.

### 3.2 NSG1 KO mice display subtle motor coordination deficits but retain gross motor function

**FIGURE 2:**
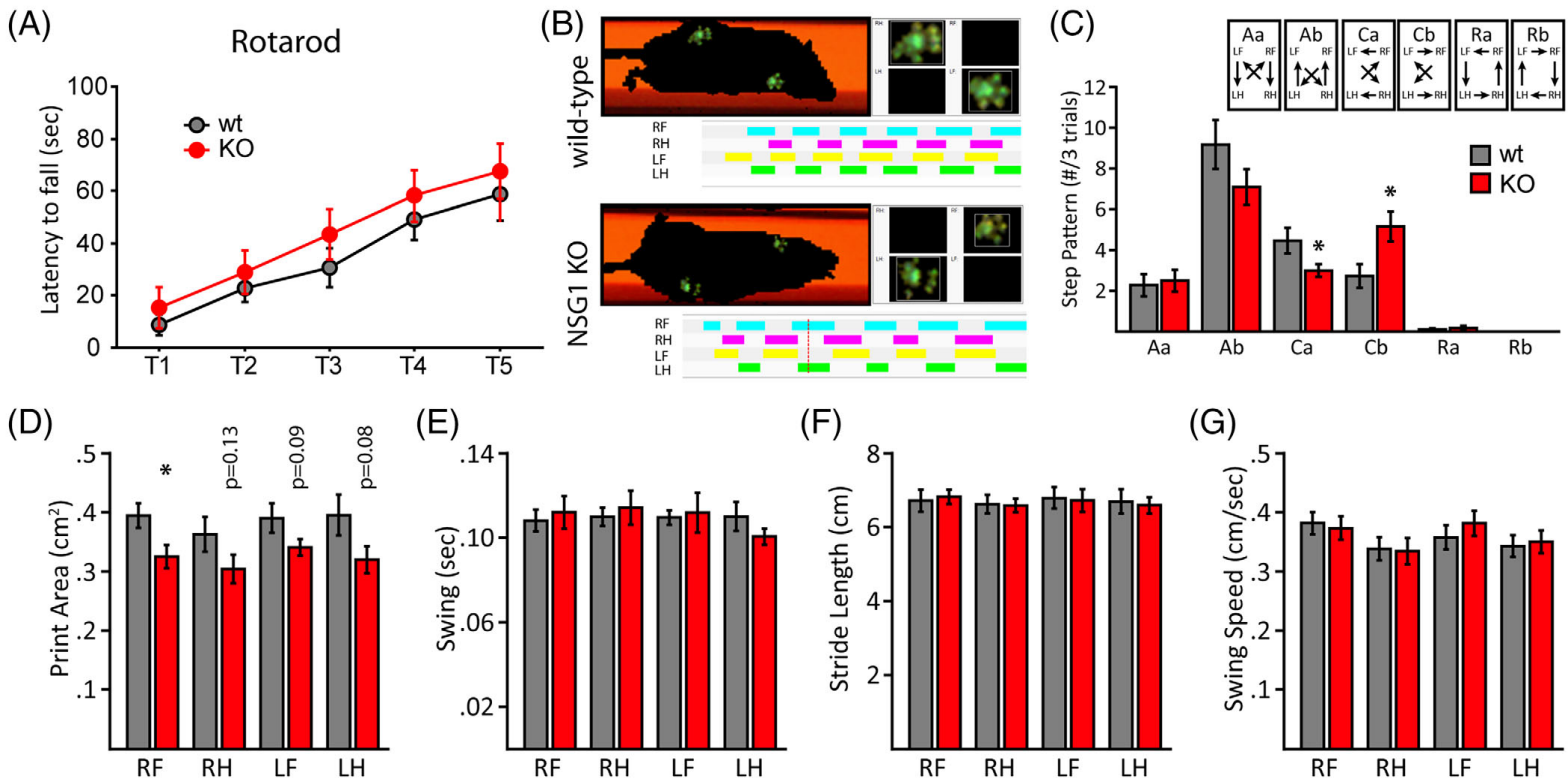
NSG1 KO affects motor coordination but not motor learning. (A) The latency to fall from the rotating rod (4–40 RPM) is presented. Means from both groups increased across trials and there were no difference between wild‐type and KO animals. Results are expressed as mean ± SEM. (B) Representative compliant runs of wild‐type (upper) and NSG1 KO (lower) animals depicting automated detection of left hind (LH), right hind (RH), left front (LF), and right front (RF) paws. (C) Pooled data reveal significant differences between genotypes specifically for crucient (Ca, Cb) step patterns but not for alternate (Aa, Ab) or rotary (Ra, Rb) step sequences. (D) Genotypes also differed significantly in total print area, with statistical significance reached for RF, but where all paws showed ~16% reduced print size. Genotypes did not differ in stride length (E), swing time (F), or swing speed (G). *n* = 12/genotype

Because of the unique expression of NSG1 in cerebellum,^7^ we first tested whether NSG1 KO animals would display motor deficits using two complementary methods. First, we used the rotarod test as a measure of gross motor impairment and learning, and predicted that NSG1 KO animals would display reduced latencies to fall off the beam during acceleration. Consistent with previous reports,^28^ we found a significant main effect of trial across groups (*F*_[4,92]_ = 27.02, *p* < 0.0001), indicating that both groups significantly increased their ability to stay on the rotating beam across trials (Figure 2A). However, there was no significant difference in the latency to fall between wild‐type and KO mice across all five trials (*F*_[1,23]_ = 1.62, *p* = 0.21). Thus, NSG1 KO animals were able to undergo motor learning equally as well as control animals.

Next we used the Catwalk XT apparatus to determine whether NSG1 KO animals display more subtle abnormalities in overall gait and locomotion. Figure 2B illustrates representative images of footfalls from control (upper panel) and NSG1 KO (lower panel) animals along with stride patterns. Figure 2C illustrates pooled data from step pattern analysis where patterns are defined as either alternating (A), crucient (C), or rotary (R) with subpatterns (a or b) illustrated in the insets. Two‐way ANOVA revealed a significant interaction between genotype and step pattern (Figure 2C; *F*_[5115]_ = 3.93, *p* = 0.002). Bonferroni post‐hoc test revealed a significant reduction in crucient step pattern (Ca; *p* = 0.04) with a concomitant increase in the complementary crucient step pattern (Cb; *p* = 0.024). In addition, we found a significant interaction between genotype and print area (Figure 2D; *F*_[3,72]_ = 4.06; *p* = 0.023), with post‐hoc tests showing significant reductions in print area for the right front (RF) paw of NSG1 KO animals (*p* < 0.05), while all other paws showed similar magnitude changes and a trend toward significance (Figure 2D). No main effects or interactions were found for swing duration, stride length, or swing speed (Figure 2E‐G; *p* > 0.05). Taken together, these data indicate that loss of NSG1 causes significant gait abnormalities but that these do not impair animals on gross motor coordination or learning compared with controls.

### 3.3 NSG1 KO animals display increased anxiety on elevated tasks

**FIGURE 3:**
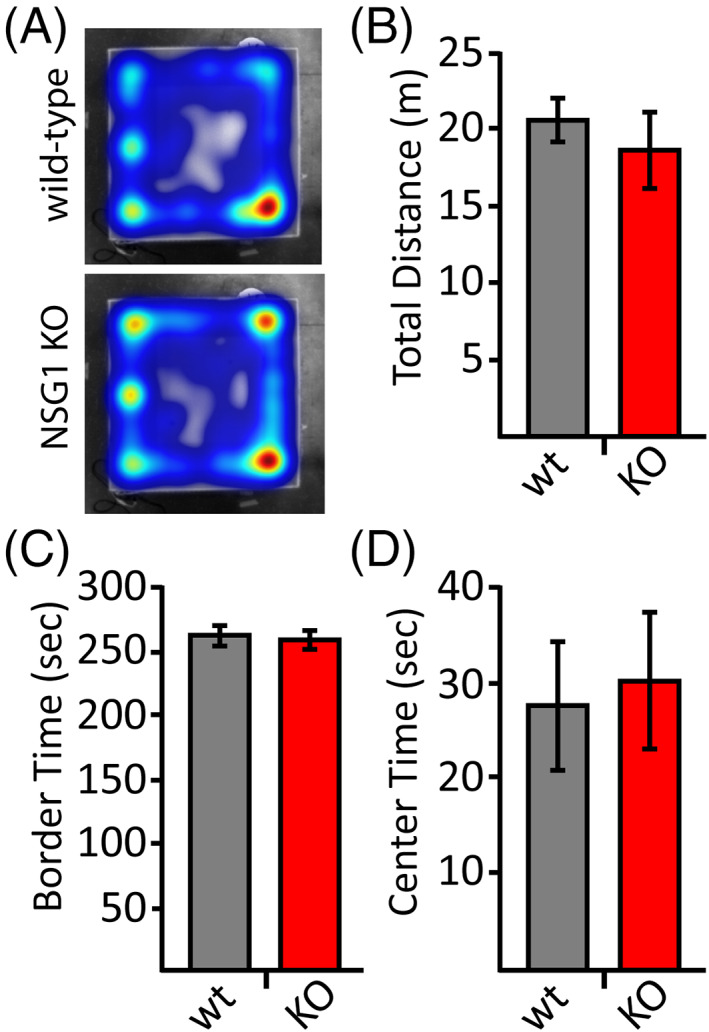
Loss of NSG1 does not cause alterations in open field exploration. (A) Representative heat maps for wild‐type (upper panel) and NSG1 KO (lower panel) animals. (B‐C) Pooled data reveal no significant differences in open field task for measures of total distance traveled (B), time spent along the border (C), nor time spent in the center (D). Results are expressed as mean ± SEM. *n* = 12/genotype

We next tested for altered affective behavior using multiple tasks designed to evoke anxiety or fear. We first used the open field test to assess general ambulation, anxiety, and exploratory behavior. NSG1 KO mice showed no differences compared with wild‐type animals in total distance traveled, suggesting there was no gross ambulation deficit in KO animals (Figure 3B: WT vs KO; *p* > 0.05), consistent with data from the rotarod task. The mutant mice also showed no difference in the time spent in the border (Figure 3A,C; WT vs. KO; *F*_[1,23]_ = 0.130; *p* = 0.72) or center of the apparatus (Figure 3A,D; WT vs. KO; *F*_[1,23]_ = 0.062; *p* = 0.81). These findings indicate that there was no difference between groups in generalized anxiety levels or exploratory behavior.

**FIGURE 4:**
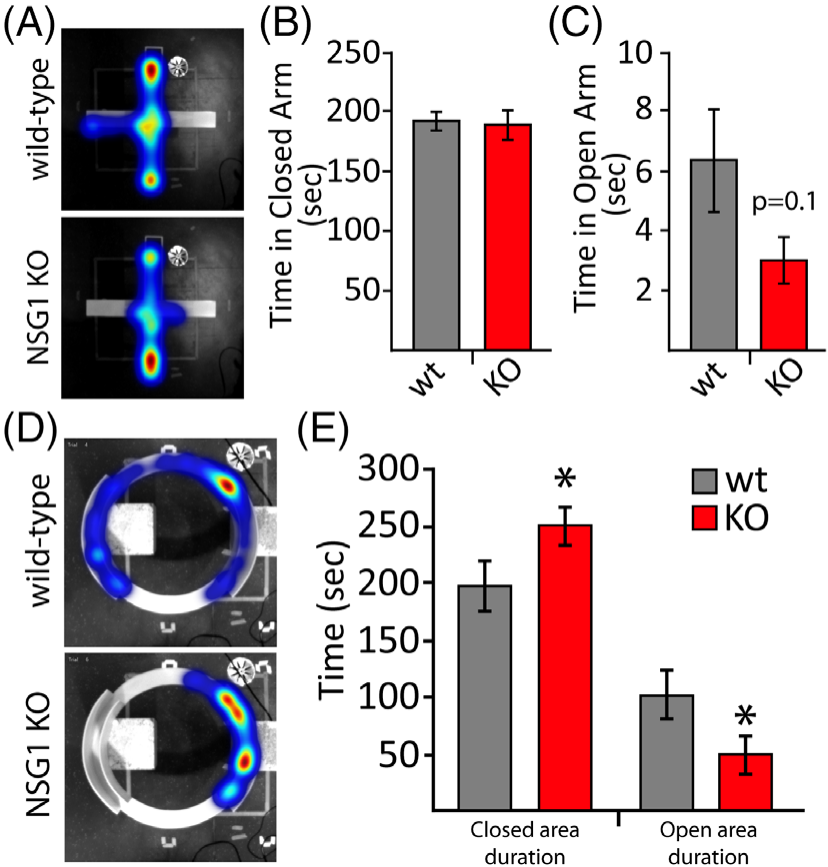
NSG1 KO results in increased anxiety‐like behavior in elevated tasks. (A‐C) Representative heat maps for wild‐type (upper panel) and NSG1 KO (lower panel) animals in the EPM task. Pooled data for time spent in the closed arms (B) and open arms (C) are presented. NSG1 mutant animals showed a twofold reduction for time spent in the open arms, although this failed to meet the a priori significance threshold of *p* = 0.05. (D) Representative heat maps for the same animals in the EZM task. (E) NSG1 KO mice spent significantly more time in the closed areas and less time in the open areas of maze (*p* < 0.05). Results are expressed as mean ± SEM. *n* = 12/genotype

We next used the elevated plus maze which takes advantage of the natural tendency of mice to avoid open areas similar to the open field task, but with the addition of the elevation element along with relatively narrow platforms. No differences were found between genotype in time spent in the closed arm (Figure 4A,B; *F*_[1,23]_ = 0.043; *p* = 0.84) or in the center of the apparatus (*F*_[1,23]_ = 0.01; *p* = 0.97). Interestingly, NSG1 KO animals displayed a mean 50% reduction in time spent in open arms, although this did not reach a statistically significant effect (Figure 4A,C; *F*_[1,23]_ = 4.698; *p =* 0.102). No differences were observed in the total distance traveled (*p* > 0.05) nor number of entries into each area (*F*_[2,69]_ = 1.66; *p =* 0.20), indicating normal activity across groups. However, together these data pointed to a possible increase in anxiety in NSG1 KO animals that was not statistically significant because of a potential floor effect given the limited time for all animals spent in the open arms.

We next used a complementary method to assess whether NSG1 KO truly displayed heighted anxiety. The EZM has been purported to reflect purely anxiety‐related behaviors by removing choice of which arms to enter and was found to be a more accurate anxiety assessment.^22^ Furthermore, animals tend to spend more time in the open areas of the EZM, reducing the likelihood of a floor effect. In this task we found no significant difference in total distance traveled (*p* < 0.05) or number of entries (*F*_[1,23]_ = 0.006; *p =* 0.99), similar to open field and EPM. However, a significant interaction effect was found between genotype and time spent in each arm (Figure 4D,E; *F*_[1,23]_ = 8.499; *p =* 0.006), with post‐hoc comparisons demonstrating a significant increase in time spent in the closed arm with a significant decrease in time spent in the open regions (Figure 4D,E; *p* = 0.04). Together, these two complementary datasets support a role for NSG1 in circuits related to stress and anxiety, whereby loss causes increased anxiety specifically in elevated tasks.

### 3.4 NSG1 KO mice do not display differences in associative learning and memory

**FIGURE 5:**
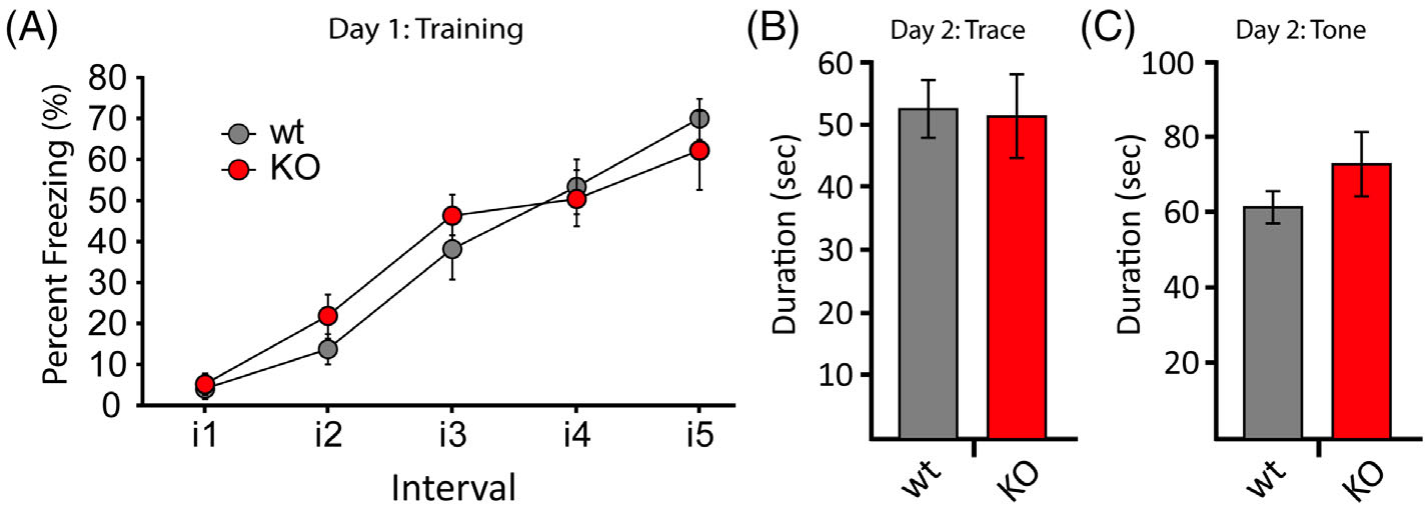
NSG1 KO animals display normal associative memory. (A) Percentage of time spent freezing is illustrated across five acquisition trials. (B) Compared with wild‐type mice, NSG1 mutants exhibited no difference in the overall percentage of time spent freezing during the trace period (B; *p* > 0.05) nor during the tone itself (C; *p* > 0.05) when presented in a novel context 24 h following training. Results are expressed as mean ± SEM. *n* = 12/genotype

Because NSG1 has been shown to be critical for the recycling of postsynaptic AMPA receptors^5^ and stable LTP,^6^ we tested KO mice on an associative memory task, thought to require proper trafficking of AMPARs within postsynaptic densities. Analysis of associative learning was assessed with the trace fear conditioning (TFC) task under different conditions. Day one training consisted of five tone‐shock pairings with 20‐s trace interval separation between the 30‐s tone and 2‐s shock. Here we found a significant main effect of trial on percentage of time spent freezing during the 30‐s tone (*F*_[4,92]_ = 59.67; *p* < 0.0001) and the trace interval (Figure 5A: *F*_[4,92]_ = 54.21; *p* < 0.0001), indicating that animals learned to associate the tone CS and trace period with the shock US. However, during training we found no significant effect of genotype on the percentage of time spent freezing across either the tone (*F*_[4,92]_ = 0.0086; *p* = 0.93) or trace (*F*_[4,92]_ = 0.0527; *p* = 0.82) interval. On the following day, mice were tested for freezing to the CS alone without the US present in a novel context. No significant main effect of genotype was found for the total percentage of time freezing for either the trace (Figure 5B: *F*_[1,23]_ = 1.196; *p* = 0.24) or tone periods in the novel context (Figure 5C: *F*_[1,23]_ = 0.0557; *p* = 0.96). Together, these data suggest that NSG1 is not critically involved in mediating hippocampal‐ or amygdala‐dependent associative memory, in contrast to previous reports suggesting involvement with hippocampal plasticity.

### 3.5 Loss of NSG1 increases activity during wake cycles

**FIGURE 6:**
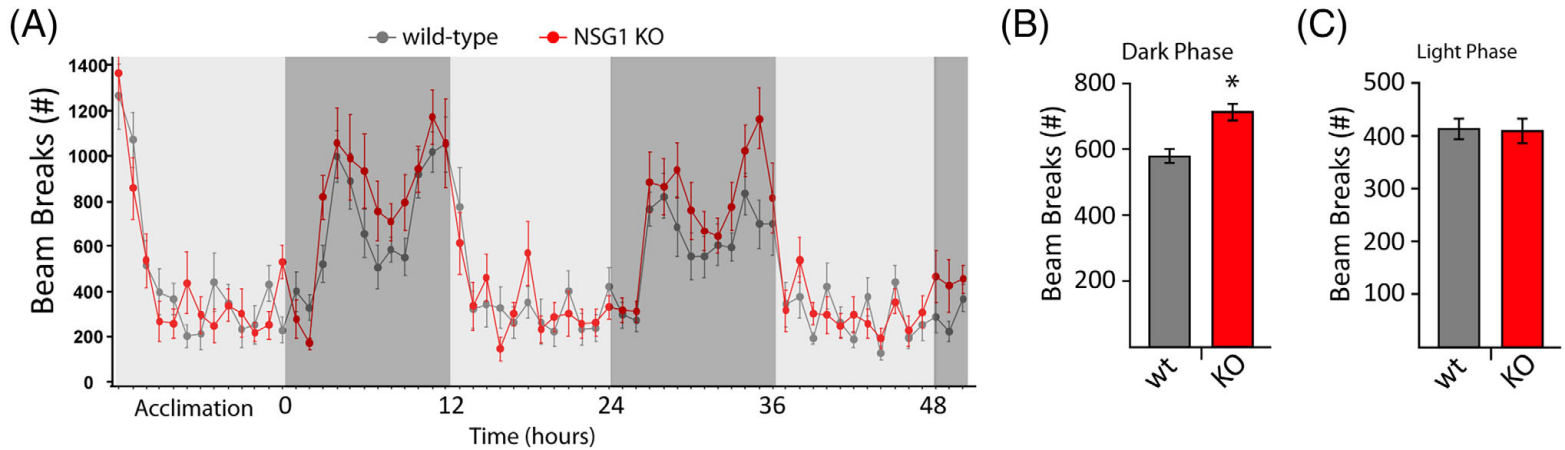
NSG1 KO selectively increases diurnal activity. (A) Homecage behavioral monitoring using photocell beam breaks to measure horizontal activity across 48 h. The first 9 h were considered acclimation and excluded from subsequent analyses. There was a highly significant effect of phase (light vs. dark) where animals showed significantly more activity during dark periods (*p* < 0.0001). (B) NSG1 KO animals were significantly more active than wild‐type animals during the dark phase (*p* < 0.05), while no difference was found for the light phase (*p* > 0.05). n = 11 controls and 12 NSG1 KOs

As sleep–wake cycles and circadian rhythms have become increasingly associated with neurological disorders, we used homecage monitoring to determine whether loss of NSG1 causes alterations in sleep–wake activity across several circadian cycles. Homecage monitoring serves as a beneficial platform for analysis of circadian locomotor activity without introducing an aversive environment like maze–based tasks. Seventy‐two hours of continuous activity monitoring were captured to analyze spontaneous activity. Two complete circadian cycles following an acclimation period were used for analysis (Figure 6A). A repeated measures ANOVA across the entire 48‐h analysis window revealed a significant interaction effect of genotype by time (*F*_(48,1008)_ = 1.38, *p* = 0.04). To clarify whether this difference was phase dependent, we separated light (sleep) and dark (wake) phases. Two‐way ANOVA found a significant effect of phase (*F*_[1,42]_ = 32.30, *p* < 0.0001) indicating mice were more active during their dark phase regardless of genotype. Bonferroni's post‐hoc comparison revealed genotypes differed significantly during the dark phase (Figure 6B; *p* = 0.04) where NSG1 KO animals displayed increased activity compared with controls. No difference in activity was seen during the light phase (Figure 6C; *p* = 0.99), indicating that loss of NSG1 did not affect activity during subjective night. Together, these data implicate NSG1 as critical for maintenance of circadian amplitude and wake activity.

## 4 DISCUSSION

Here we demonstrate that while KO of NSG1 does not significantly impact gross motor or cognitive function, NSG1 KO affects anxiety‐related behaviors, motor coordination, and circadian amplitude but not phase durations. Interestingly, some results did not generalize across modalities such as anxiety‐related behavior. For instance, NSG1 KO animals performed similarly in the open field test to wild‐type counterparts, showing equivalent time in the center of the apparatus, an area generally not preferred by rodents. However, in more anxiety‐inducing tests such as the elevated plus and zero maze, NSG1 KO animals showed heightened anxiety‐like behaviors, and reduced time spent in the open regions.

As NSG1 is uniquely expressed in cerebellar Purkinje neurons among NSG family members,^7^ we were particularly interested in its importance for motor function. While we did not observe significant deficits on gross motor function or learning on the rotarod task, we saw significant alterations in gait and coordination. These took the form of overall decreased paw print size as well as altered step patterns, which are observed in genetic insults that cause cerebellar dysfunction.^29^, ^30^ This may be a critical component of the heightened anxiety phenotype observed on elevated tasks as the combination of the open (exposed) arms of the EPM and Zero Maze converge on circuitry that require NSG1 for normal function. Unlike the open field task that presents no overtly threatening stimuli (e.g., elevation), EPM and EZM may uniquely recruit circuits that respond to this type of anxiety‐inducing environment. Despite lack of gross motor deficits, it may be the case that NSG1 animals have subtle cerebellar dysfunction related to elevation or general sense of unsteadiness that converges on anxiety‐related circuits. Evidence in human and primate studies have found that cerebellar circuits, especially those in the posterior cerebellum, are critical for nonmotor tasks, and may be involved in the processing of emotion. Lesions of the cerebellar vermis alter fear responses by decreasing freezing and increasing open field exploration.^31^ On the other hand, stimulating the vermis induces fear responses, such as increased amplitude of the acoustic startle response.^32^ Future studies using the conditioned eyeblink task across multiple NSG family member KO mice (e.g., NSG2‐3), could help determine whether the cerebellum may be a locus for anxiety‐related behavioral changes specifically in NSG1 KO mice.

The current model of NSG‐mediated AMPAR trafficking within dendritic spines suggests a primary role of NSG3 (Calcyon) with the endocytosis of surface AMPARs, and NSG1 with recycling of internalized AMPARs back to the plasma membrane during plasticity‐inducing stimuli.^8^ Thus, their roles can largely be thought of as opposing one another in terms of promoting AMPAR surface expression. In this way, it may be predicted that NSG1 KO may phenocopy the behavioral performance of animals that overexpress NSG3/Caly. Interestingly, NSG3 OE animals do display similar phenotypes as NSG1 KO across motor and anxiety‐related domains. For instance, NSG3 OE animals display spontaneous hyperactivity when exploring an open field apparatus in addition to stereotypies, rearing and sniffing behaviors.^15^ Whereas NSG1 animals did not show increased total distance traveled in the open field, they do show increased overall activity during homecage monitoring, suggesting hyperactivity at least in specific contexts. Our open field task only assessed behavior for 5 min, which may have been inadequate to detect differences in activity since mice generally spend the first few minutes relatively inactive in a novel context. Perhaps this is why extended homecage monitoring was able to detect this difference.

While activity data support the inverse roles of NSG1 and NSG3, data from other tasks appear to contradict this model. For instance, NSG3 OE animals displayed reduced anxiety, spending more time in the light areas of a light–dark box.^15^ In contrast, NSG1 KO animals showed enhanced anxiety in similar tasks (EPM/EZM). Furthermore, the impacts of NSG1 loss compared with NSG3 OE appear to diverge with respect to hippocampal‐dependent learning and the molecular mechanisms for these circuits. Animals with NSG3 OE displayed enhanced, perseverative freezing in fear conditioning tests as well as perseverative exploration in Morris water maze when platform location was changed.^13^ In contrast, NSG1 is dispensable for hippocampal‐dependent associative learning according to our data. All of these behaviors are largely dependent on processes thought to involve alterations in post‐synaptic AMPARs.^33^ As mentioned, NSG1 is thought to play a primary role in AMPAR recycling following potent NMDA receptor activation or those that cause LTP.^5^, ^6^ However, recent data by Yap and colleagues found robust colocalization between NSG1 and NSG2 in early and late endosomes, but not robust localization with Rab11^+^ recycling endosomes.^10^ Further, our recent findings place NSG2 at a relative minority of synapses,^9^ which predicts that NSG1 may lack universal synaptic localization as well. Regardless, the robust colocalization of NSG1 and NSG2,^10^ along with data showing that overexpression of NSG2 increased synaptic strength,^9^ suggest a possible compensatory effect of NSG2 in NSG1 KO animals. Our current data argue that NSG2 alone may be sufficient to promote AMPAR exocytosis to synapses under plasticity‐inducing conditions which may occur via a distributed set of vesicular compartments.^34^ Thus, future studies should revisit the involvement of NSG1 in post‐synaptic AMPAR regulation in hippocampal plasticity and examine whether NSG2 has a similar or disparate role in AMPAR recycling and exocytosis.

NSG family members are involved in a number of other cellular processes in addition to AMPAR trafficking during synaptic plasticity including trafficking of L1CAM,^2^ Neuregulin,^35^ Neurotensin receptors 1–2,^3^ as well as β‐APP.^4^ Furthermore, while NSG1 and NSG2 appear to be expressed at similar levels to NSG3 in adults, they show their highest expression during development.^36^, ^37^ Thus, additional factors need to be incorporated into models of how they affect overall circuit function. Regardless of mechanism, future studies should examine whether NSG1 OE or NSG3 KO produce the opposite results in more complex cognitive tasks of learning, memory and flexibility to see what circuits utilize NSG1 and NSG3 in a convergent or divergent manner. In addition, we recently showed that NSG2 promotes synaptic strengthening,^9^ potentially by promoting AMPAR insertion into a subset of post‐synaptic specializations. Thus, future studies using NSG2 single KO and NSG1/NSG2 double mice should inform whether behavioral abnormalities are because of the interaction of both proteins on plasticity‐induced changes to AMPAR surface expression at a subset, or all synapses. In addition, although sex differences have not been published for expression or function of NSG1/2, future studies should examine whether female animals show similar or unique phenotypes following KO.

## CONFLICT OF INTEREST

The authors declare that there is no conflict of interest that could be perceived as prejudicing the impartiality of the research reported.

## Data Availability

The data that support the findings of this study are available from the corresponding author upon reasonable request.
